# Experimental study on foaming characteristics and microstructure of self-expanding polymer grouting material under low-temperature conditions

**DOI:** 10.1371/journal.pone.0311898

**Published:** 2025-06-04

**Authors:** Songtao Li, Baolin Wang, Yanlong Gao

**Affiliations:** 1 College of railway engineering, Zhengzhou Railway Vocational & Technical College, Zhengzhou, China,; 2 School of Water Conservancy and Transportation, Zhengzhou University, Zhengzhou, China,; 3 Henan Jiaoyuan Engineering Technology Group Co., Ltd, Zhengzhou, China; Shandong University of Technology, CHINA

## Abstract

Polymer grouting materials, due to their self expansion properties, have been widely used in repair engineering involving hydraulic structures and have become a new type of building material. The foaming characteristics of polymer grouting materials under low-temperature conditions directly influence their repair effectiveness in cold areas. In this study, a grouting test of the free expansion of the polymer grouting material in the vertical direction and a mold grouting test with all four sides constrained were performed under different low-temperature conditions. The structure and morphology of foam cells under different low-temperature conditions were examined via scanning electron microscopy. The test results indicated that as the temperature decreased, the volume of the foam gradually exhibited a cold shrinkage effect, and the surface temperature gradually decreased. For every 1°C decrease in temperature, the foaming volume decreases by an average of 40 cm^3^, and the surface temperature decreases by an average of 1.7°C. According to the foaming rate, the foaming process was divided into three stages: fast, slow, and stable foaming. Additionally, the internal temperature stress generated during the foaming process was a key factor that caused the foam to bulge outward. Under the condition where all four sides were constrained, the density of the foam formed gradually decreased as the temperature decreased. The foam cell was spherical. As the temperature decreased, the cell morphology became blurred, and the number of incomplete cell structures between the gaps increased. The research results will provide a reference for the polymer repair projects of hydraulic structures in cold regions.

## Introduction

In the repair process using subgrade grout technology, cement concrete grout or polymer chemical grout is injected into the loose soil of the subgrade through a grouting pipe. This technology eliminates the need for large-area excavation and replacement of subgrades and is currently one of the main methods for repairing loose soils in subgrades [1,2]. Compared with cement grouting technology, the most significant advantage of the polymer grouting material is that it is a self-expanding polymer chemical slurry that can generate high-strength foam through the polymerization reaction between components. Thus, the loose soil can be bonded and filled for strengthening the roadbed [3–5].

Numerous studies have been performed on the properties of polymer grouting materials. Hao et al. studied the diffusion shape of the polymer grouting material in the cracks of a plate through experiments and a numerical simulation analysis and conducted a theoretical analysis of the rheological properties of the polymer. According to the research results, a density model suitable for polymer slurry diffusion was established [6]. Liu et al. performed a large-scale model test of central drawing to examine the bonding characteristics between soil and a polymer grouting material of different densities. From a microscopic viewpoint, the bonding mechanism between the polymer grouting material and the soil was analyzed, and the distribution law of the bonding force between the polymer and the critical surface of the soil was obtained [7]. Shi et al. studied the temperature changes during the foaming process of a polymer grouting material through a self-developed grouting mold and believed that the foaming process of polymer slurries was divided into three stages: heating, constant temperature, and cooling [8]. Cao et al. used an electron microscope scanner to examine the microstructure of an improved polyurethane polymer grouting material [9].

Additionally, polymer grouting materials have been used in engineering applications. Guo et al. studied the mechanical response of the lower voids of airport pavement filled with a polymer grouting material and concluded that this method is a quick and practical repair method [10]. Fang et al. measured the mechanical parameters of a polymer grouting material indoors and studied the dynamic stress changes of polymer grouting materials during the process of raising railway foundations through numerical simulation methods. The results indicated that the polymer grouting material can quickly raise the railway subgrade, preventing the long-term suspension of the high-speed rail due to subgrade maintenance [11]. Cui et al. used a polymer grouting material damage constitutive model to study the fatigue damage of the polymer grouting material and the cement grouting material filling the lower voids of the cement concrete pavement and believed that the polymer grouting is more effective for repair than the cement grouting [12]. Wang et al. investigated the leakage of underground drainage pipes, analyzing the mechanical changes of the polymer grouting used to repair the seepage damage of the pipe under different soil cover layer thicknesses and load types. The results indicated that the polymer grouting can effectively repair broken pipes and even restore their mechanical properties to those of normal pipes [13].

In summary, polymer grouting technology has been widely used in the restoration of structures in non-cold areas. The polymer grouting material is a polymer chemical slurry with two raw materials. The formation of high-strength foam requires a polymerization reaction between two raw materials, and the degree of this polymerization reaction significantly affects the foaming performance of the polymer grouting material [14–16]. Research has indicated that temperature changes generally affect the energy of the reactant molecules, which is one of the important factors influencing the rates of the chemical reactions. When the temperature increases, the molecules gain energy, increasing the rates of the chemical reactions. When the temperature decreases, some molecules lose energy, which reduces the chemical reaction rates [17,18].

In the process of engineering repair in cold regions, the foaming performance of the polymer grouting material under low-temperature conditions directly influences the repair effect. Therefore, research on the foaming performance of polymer grouting materials under low-temperature conditions has considerable engineering significance. In this study, the free foaming shape of the foam in the vertical direction was described under different low-temperature conditions, and the generation conditions of the temperature stress and the mechanism of the influence on the foam shape were analyzed. Then, using a self-developed iron grouting mold, foam specimens with different grouting amounts were prepared, and the uniformity of the foam density under the condition where all four sides were constrained was analyzed. Finally, the microstructures of the foam cells were examined via SEM. In this study, methods ranging from the microscale to the macroscale were adopted to investigate the effects of the temperature on the foaming characteristics of the polymer grouting material to provide a reference for the repair of cold areas.

## Materials and methods

### Materials

**Fig. 1:**
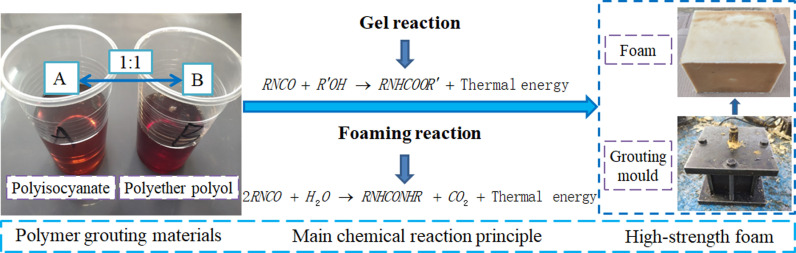
Polymer grouting material and reaction principle.

Polyurethane polymer grouting material is mainly composed of polyisocyanate, polyether polyol, a foaming agent, a catalyst, a foam stabilizer, and various other additives.The self-expanding polymer grouting material used in the study is a polymer chemical slurry composed of raw materials A and B. The main component of material A is polyisocyanate, and the main component of material B is polyether polyol. After the two raw materials are mixed at a ratio of 1:1, the main chemical reactions that occur under the action of the catalyst are the gel reaction and the foaming reaction, and finally a high-strength foam is formed. The polymer grouting material and reaction principle are shown in Fig 1. The gel curing reaction occurs between the polyisocyanate -NCO groups and the polyol -OH groups; the foaming reaction results from the reaction of the polyisocyanate -NCO groups and H_2_O to form CO_2_ and urea.

#### Test equipment.

The polymer grouting integrated system used in the test includes a pressure-supply device, a grouting pipe, a grouting gun, and a toolbox for cleaning the grouting equipment regularly. During the grouting test, the polymer grouting material is drawn into the pressure supply device through the conveying slurry pipe. Then, it is conveyed into the grouting pipe under pressure, and the high-pressure polymer slurry is sprayed by the grouting gun. The polymer grouting test was performed outdoors, and the outdoor temperature was an uncontrollable factor. Therefore, to realize the polymer grouting test under different low-temperature conditions, a refrigerator was used to simulate the low-temperature environment [19,20]. The temperature range of the refrigerated box used in the test was –40 to 20°C, for simulating different low-temperature environments.

**Fig 2:**
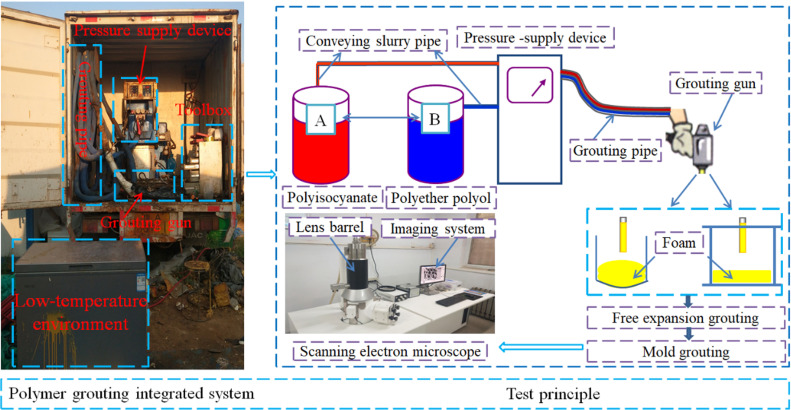
Test equipment and principle.

The microstructures of the foam formed under different temperature conditions were examined via scanning electron microscopy (SEM, KYKY 6200). A highly focused electron beam was used to scan the samples and obtain various physical data. The data were processed by a computer processor and magnified and imaged on the display to determine the microscopic features of the test sample surface [21,22]. The test equipment and principle are shown in Fig 2.

#### Test method and process.

According to the law of environmental temperature changes in cold regions [23–25], the low-temperature environmental temperature gradients selected in this study were –20, –10, –5, 0, and 5°C. The polymer grouting test was divided into two processes: free expansion grouting in the vertical direction and mold grouting with all four sides constrained.

**Fig 3:**
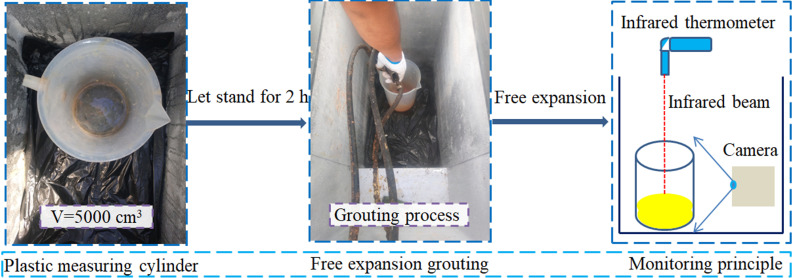
Free expansion grouting test process.

The process of the free expansion grouting test in the vertical direction was as follows. First, the temperature of the low-temperature environment chamber was adjusted to the test standard temperature, and a plastic measuring cylinder cup with a capacity of 5000 mL was placed in the low-temperature environment chamber for 2 h. Then, the integrated polymer grouting system was activated, and 250 g of polymer grouting material was evenly injected into the measuring cylinder. Finally, the whole foaming process was recorded using a video camera, and the temperature change of foam surface was measured using an infrared thermometer. The test scheme is presented in Table 1, and the test process is shown in Fig 3.

**Table 1:** Free expansion grouting test scheme.

Test number	1	2	3	4	5
Temperature/°C	–20	–10	–5	0	5
Grouting mass/g	250	250	250	250	250

**Fig 4:**
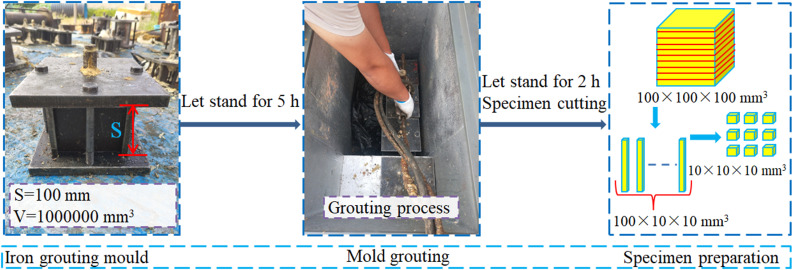
Mold grouting test process.

The process of the mold grouting test with all four sides constrained was as follows. The self-developed iron grouting mold with a size of 100 mm ×  100 mm ×  100 mm was placed in a low-temperature environment warehouse for 5 h. Then, the polymer grouting material (with masses of 200, 300, 400, and 500 g) was injected into the mold by the grouting gun and left to stand for 2 h. After the demolding of the test piece, a cutting machine was used to cut from top to bottom to obtain a rectangular parallelepiped (100 mm ×  10 mm ×  10 mm) for testing the uniformity of the density. Finally, the 300-g grouting test piece was cut into 10 mm ×  10 mm ×  10 mm cubes for SEM characterization of the foam particles under different density and temperature conditions. The test scheme is shown in Table 2, and the test process is shown in Fig 4.

**Table 2:** Mold grouting test scheme.

Test number	1	2	3	4	5
Temperature/°C	–20	–10	–5	0	5
Grouting mass/g	200	200	200	200	200
	300	300	300	300	300
	400	400	400	400	400
	500	500	500	500	500

## Results and discussion

### Free volume expansion law of foam

**Fig 5:**
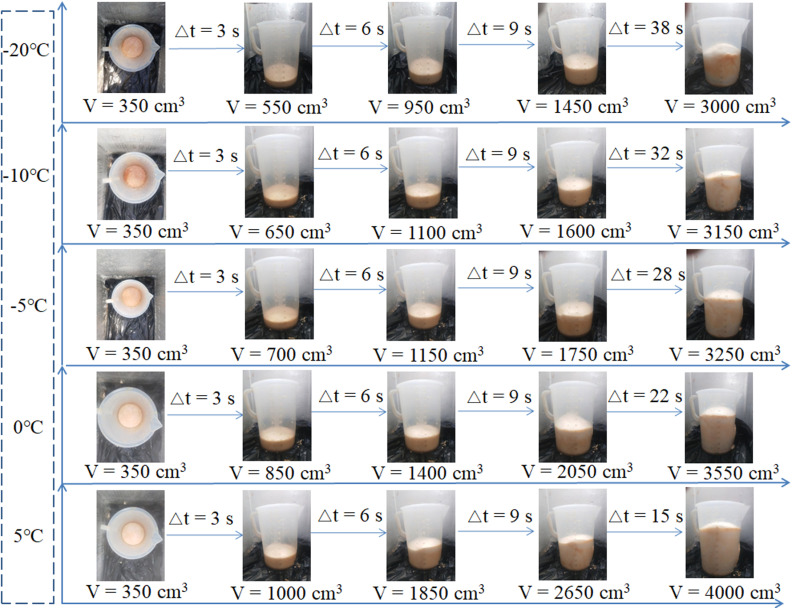
Volume expansion of the foam.

To vividly describe the free foaming process of the polymer grouting material at different temperatures, the foam shapes at different times were photographed, as shown in Fig 5. When the temperature was –20°C, the foaming time of the polymer grouting material was 38 s, and the foaming volume was 3000 cm^3^. When the temperature was –10°C, the foaming time was 32 s, and the foaming volume was 3150 cm^3^. When the temperature was –5°C, the foaming time was 28 s, and the foaming volume was 3250 cm^3^. When the temperature was –0°C, the foaming time was 22 s, and the foaming volume was 3550 cm^3^. When the temperature was 5°C, the foaming time was 15 s, and the foaming volume was 4000 cm^3^. The test results indicate that in the early stage of foaming, a lower temperature corresponded to a smaller foam volume. Additionally, as the temperature decreased, the foaming time of the polymer grouting material increased. Thus, the temperature reduction affected the foaming reaction of the polymer grouting material, resulting in the shrinkage of the foam.

**Fig 6:**
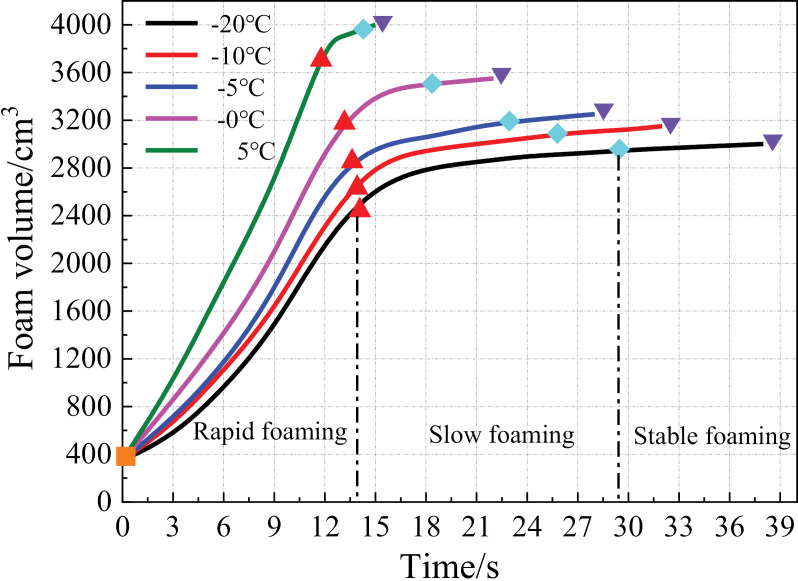
Volume variations of the foams.

Fig 6 presents the linear change rule of the foam volume with respect to time under different temperature conditions. As shown, the foaming rate of the polymer grouting material gradually decreased with a reduction in the temperature. Under the same temperature conditions, the foaming process was divided into three stages according to the foaming rate: rapid, slow, and stable foaming. In the rapid foaming stage, after the two raw materials A and B were sprayed by the grouting gun, they quickly merged, and a violent chemical reaction occurred, resulting in rapid volume expansion. In the slow foaming stage, owing to the loss of raw materials, the intensity of the chemical reaction was reduced, and the volume expansion rate began to decrease. Compared with the two aforementioned stages, the foaming rate in the stable foaming stage was significantly reduced, but the volume increased nonetheless. Additionally, the durations of the three foaming stages all increased with the decreasing temperature, and the increase was relatively large for the slow and stable foaming stages.

### Law of surface temperature change

**Fig 7:**
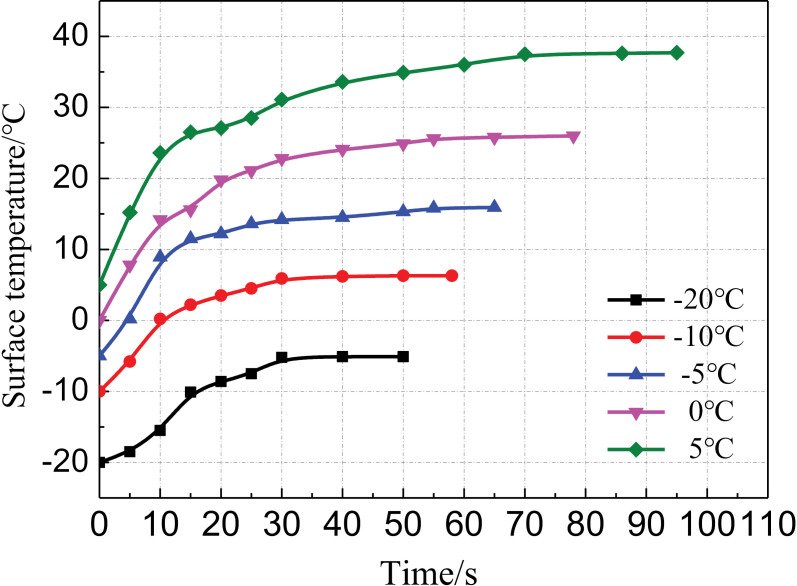
Change law of the foam surface temperature.

Fig 7 presents the change law of the surface temperature of the foam under different temperature conditions. As shown, the maximum temperature of the foam surface decreased with the decreasing ambient temperature. At the ambient temperature of –20°C, the maximum temperature of the foam surface was only –4.5°C. At the ambient temperature of 5°C, the maximum temperature of the foam surface was 38°C. A lower ambient temperature corresponded to a shorter increase time for the surface temperature of the foam. In the early stage, the surface temperature of the foam increased rapidly. However, in the later stage, the surface temperature exhibited slow growth. The results indicate that the surface temperature of the foam was related to not only the exothermic heat of the chemical reaction between the surface materials but also the outward transfer of heat from the inside of the foam.

**Fig 8:**
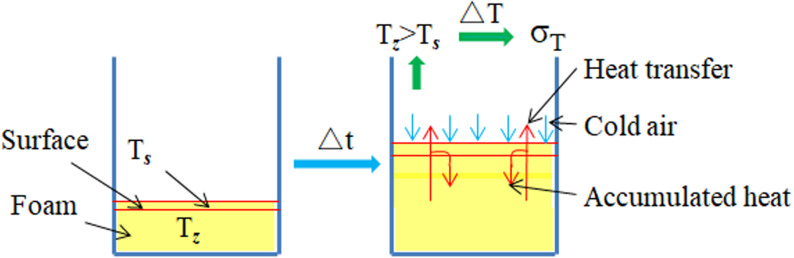
Generation of temperature stress.

The test phenomena are explained in Fig 8. Here, T_*s*_ represents the surface temperature of the foam, T_***z***_ represents the center temperature of the foam, and σ_T_ represents the temperature stress. In the early stage of the foaming reaction, the chemical reaction between the two raw materials A and B began. At this time, T_*s*_ and T_***z***_ were almost equal. After a short period of time, a wrapper was formed on the surface of the foam. The wrapper hindered the transfer of heat inside the foam, and part of the heat was collected inside the foam. Coupled with the cooling effect of the outside cold air, a temperature difference was gradually formed between T_***s***_ and T_***z***_, resulting in temperature stress. As the foaming process continued, this temperature difference increased, increasing the temperature stress.

### Effect of temperature stress on morphology of foam

**Fig 9:**
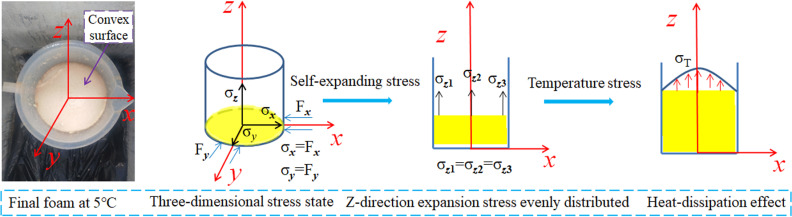
Surface bulging principle of the foam.

As shown in Fig 5, the final shape of the foam surface was convex. This indicates that the temperature stress in the vertical direction affected the morphology of the foam. To analyze the cause of this test phenomenon, a three-dimensional rectangular coordinate system was established with the center of the foam bottom surface as the origin, as shown in Fig 9.

The inner wall of the measuring cylinder used in the test was evenly coated with lubricating oil and assumed to be in a smooth state. Therefore, the frictional force generated by the inner wall of the measuring cylinder against the foam was ignored. The volume expansion force produced by the polymer grouting material during the foaming process can be expressed by formula (1).


$$\sigma_{x}=\sigma_{y}=\sigma_{z}$$ (1)

Where $\sigma_{x}$,$\sigma_{y}$, and $\sigma_{z}$represent the self-expansion stresses of the foam in the *x*, *y*, and *z* directions, respectively. According to Newton’s third law, if there is no external force, when the volume expansion force in the *x* and *y* directions acts on the inner wall of the measuring cylinder, the inner wall produces equal and opposite reaction forces.


$$\sigma_{x}=F_{x}=\sigma_{y}=F_{y}$$ (2)

Where, $F_{x}$and $F_{y}$represent the binding forces of the inner wall of the measuring cylinder in the *x* and *y* directions, respectively. Therefore, the foam can only expand (in volume) along the *z*-axis direction. Because the friction generated by the inner wall of the measuring cylinder is ignored, the volume expansion forces of the foam at the center and the edge of the measuring cylinder are equal. In this case, the foam surface should be flat. This conclusion is consistent with the test results shown in Fig 5.


$$\sigma_{z1}=\sigma_{z2}=\sigma_{z3}$$ (3)

Where, $\sigma_{z1}$and $\sigma_{z2}$represent the self-expansion stresses on both sides of the foam in the *z* direction, and $\sigma_{z3}$represents the self-expansion stress of the center of the foam in the *z* direction. Over time, the raw materials of the polymer grouting are gradually lost, and the *z*-direction self-expansion stress of the foam gradually decreases. When the volume expansion stress in the *z* direction cannot satisfy the volume expansion, the temperature stress generated inside the foam causes the foam to continue its volume expansion along the positive direction of the *z* axis. However, because the temperature stress generated in the center of the foam is greater than the boundary temperature stress, the surface of the foam finally bulges outward.

### Effect of low temperature on density uniformity of foam

**Fig 10:**
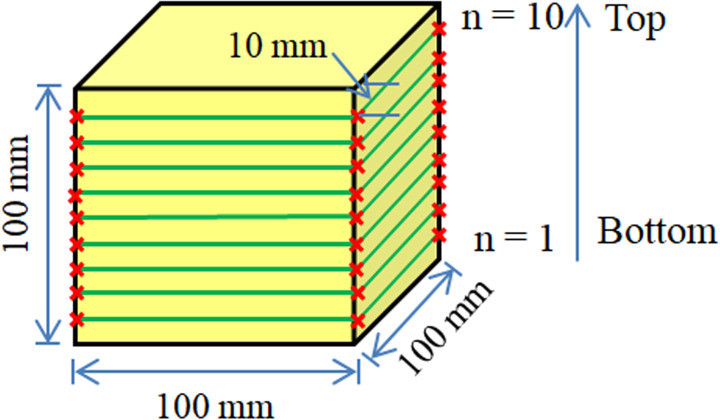
Cutting diagram of the foam.

**Fig 11:**
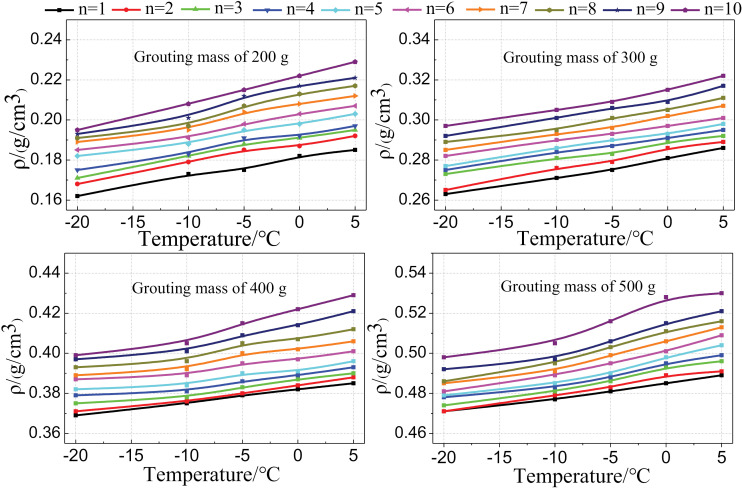
Density uniformity of the foam.

Density is one of the most important parameters for evaluating the strength of polymer grouting materials [26]. To analyze the uniformity of the density of the foam formed by the mold grouting, the 100 mm ×  100 mm ×  100 mm cube foam specimens made in the experiment were cut from bottom to top into 10 rectangles with a size of 100 mm ×  10 mm ×  10 mm. Then, the density calculations were performed, as shown in Fig 10. The calculation results are presented in Fig 11; as shown, under the same temperature condition, the foam density gradually increased from the bottom to the top, and a lower temperature corresponded to a smaller increase. Under the same grouting volume, the foam density at the same position decreased as the temperature decreased.

The foregoing test results indicate that the density of the foam formed in the mold was non-uniform. Owing to the self-expansion characteristics of the polymer grouting material, the foam underwent volume expansion in the vertical direction with the restriction of the side. When the volume expanded to the top of the mold, the volume of the foam no longer expanded, owing to the constraint of the steel plate on the top of the mold. However, the foaming reaction continued. Therefore, before the strength was fully developed, the foam was subjected to a downward reaction force from the top of the abrasive tool, increasing the density. At this time, because the foaming reaction at the bottom of the mold had been proceeding for a considerable amount of time, the strength of the bottom foam was higher than that of the top foam. Therefore, the compression effect of the top foam was more significant, and the density was higher at the top than at the bottom. According to the test results shown in Fig 5, under low-temperature conditions, the foam volume exhibited a cold shrinkage effect, resulting in a decrease in the volume expansion rate. Therefore, the density of the foam formed by the mold grouting decreased as the temperature decreased.

### Effect of low temperature on cell structure of foam

**Fig 12:**
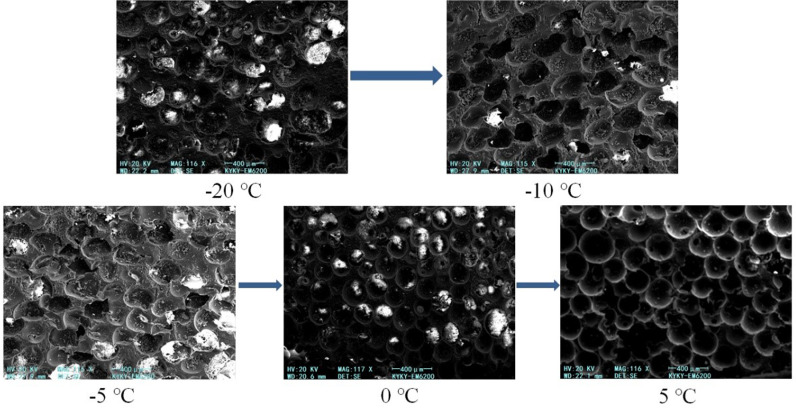
Microscopic characterization of foam cells under different temperature conditions.

When the grouting mass was 300 g, SEM images of the foam cells under different temperature conditions with a magnification of 115 × were obtained, as shown in Fig 12. At the temperatures of –20 and –10°C, the foam cells had a fuzzy shape, a relatively broken structure, large pores between them, and relatively thin cell walls. At –5°C, the shape of the foam cells was close to spherical, but the structure was still relatively broken, and there were incompletely formed cell structures between the pores. At 0°C, the overall cell shape was spherical, with thinner cell walls, a denser distribution, and fewer incompletely formed cell structures between the pores. At 5°C, the spherical cell structure was more obvious, the cell wall was thickened, the pores between the cells were smaller, and the cell size was larger than those for the other temperature conditions. The test results indicate that the foam cells were spherical. As the temperature decreased, the cell shape became blurred, the number of pores between the cells increased, and the number of incompletely formed cell structures increased.

## Conclusions

Research methods ranging from the microscale to the macroscale were used to investigate the foaming characteristics of self-expandable polymer grouting materials under different low-temperature conditions. According to the test results, conclusions are drawn.

(1) Reducing the temperature affected the foaming reaction of the polymer grouting material, resulting in the volume shrinkage of the foam. Under the same temperature conditions, the foaming process was divided into three stages: fast, slow, and stable foaming. The durations of these stages all increased with the decreasing temperature, and the increase was relatively large for the slow and stable foaming stages.
(2) The maximum temperature of the foam surface decreased as the ambient temperature decreased. The temperature stress generated inside the foam caused the foam to expand in volume along the positive direction of the *z*-axis. Thus, the surface of the foam bulged outward.
(3) Under the four-sided confinement condition, the foam density gradually increased from the bottom to the top under the same temperature condition, and a lower temperature corresponded to a smaller increase. Under the same grouting volume, the foam density at the same position decreased as the temperature decreased.
(4) The foam cells were spherical. With a reduction in the temperature, the cell shape became blurred, the number of pores between the cells increased, and the number of incompletely formed cell structures increased.
(5) The research results will provide a reference for the polymer repair projects of hydraulic structures in cold regions. For example, it can provide guidance for grouting parameters such as grouting quantity and preheating temperature of restoration projects in cold areas.
